# Oxidation and interaction of DJ-1 with 20S proteasome in the erythrocytes of early stage Parkinson’s disease patients

**DOI:** 10.1038/srep30793

**Published:** 2016-07-29

**Authors:** Yoshiro Saito, Yoko Akazawa-Ogawa, Akihiro Matsumura, Kazumasa Saigoh, Sayoko Itoh, Kenta Sutou, Mayuka Kobayashi, Yuichiro Mita, Mototada Shichiri, Shin Hisahara, Yasuo Hara, Harutoshi Fujimura, Hiroyuki Takamatsu, Yoshihisa Hagihara, Yasukazu Yoshida, Takao Hamakubo, Susumu Kusunoki, Shun Shimohama, Noriko Noguchi

**Affiliations:** 1 Systems Life Sciences laboratory, Department of Medical Life Systems, Faculty of Life and Medical Sciences, Doshisha University, Kyotanabe, Kyoto 610-0394, Japan; 2 National Institute of Advanced Industrial Science and Technology (AIST), Ikeda, Osaka 563-8577, Japan; 3 Department of Neurology, School of Medicine, Sapporo Medical University, Sapporo 060-8556, Japan; 4 Department of Neurology, Kinki University Faculty of Medicine, Osaka 589-8511, Japan; 5 Hamamatsu Pharma Research Inc., Hamamatsu 431-2103, Japan; 6 Hara Clinic, Ikeda, Osaka 563-0025, Japan; 7 Department of Neurology, National Hospital Organization Toneyama National Hospital, Toyonaka, Osaka 560-8552, Japan; 8 Laboratory of Systems Biology and Medicine, Research Center for Advanced Science and Technology, University of Tokyo, Tokyo 153-0041, Japan

## Abstract

Parkinson’s disease (PD) is a progressive, age-related, neurodegenerative disorder, and oxidative stress is an important mediator in its pathogenesis. DJ-1, the product of the causative gene of a familial form of PD, plays a significant role in anti-oxidative defence to protect cells from oxidative stress. DJ-1 undergoes preferential oxidation at the cysteine residue at position 106 (Cys-106) under oxidative stress. Here, using specific antibodies against Cys-106-oxidized DJ-1 (oxDJ-1), it was found that the levels of oxDJ-1 in the erythrocytes of unmedicated PD patients (n = 88) were higher than in those of medicated PD patients (n = 62) and healthy control subjects (n = 33). Elevated oxDJ-1 levels were also observed in a non-human primate PD model. Biochemical analysis of oxDJ-1 in erythrocyte lysates showed that oxDJ-1 formed dimer and polymer forms, and that the latter interacts with 20S proteasome. These results clearly indicate a biochemical alteration in the blood of PD patients, which could be utilized as an early diagnosis marker for PD.

*DJ-1* is implicated as the causative gene of a familial form of Parkinson’s disease (PD), namely *PARK7*, and it plays an important role in anti-oxidative defence, protecting cells from oxidative stress^1^,^2^. DJ-1 is a multifunctional protein that is involved in various physiological processes, including transcriptional regulation, mitochondrial function, and signal transduction^1^,^3^,^4^. Mutations of *DJ-1* in *PARK7* cause the loss of DJ-1 function, and increase the sensitivity to oxidative stress-induced cell death^1^,^2^,^3^,^4^. DJ-1 regulates the function of transcriptional factors such as NF-E2-related factor 2 (Nrf2) and p53, and also changes glutathione (GSH) metabolism and the expression levels of heat shock proteins (HSPs) and uncoupling proteins (UCP4 and UCP5)^5^,^6^,^7^,^8^. Furthermore, DJ-1 is known to regulate signal transduction related to oxidative stress response through an interaction with signal mediators such as PTEN and ASK1^9^,^10^,^11^. The anti-oxidative function exhibited by DJ-1 prevents oxidative stress-induced cell death by regulating transcriptional factors and signal mediators. DJ-1 acts as a redox-activated chaperone, which might account for the identification of the many DJ-1-interacting proteins described above^12^. Recently, DJ-1 was identified as a regulator of 20S proteasome^13^.

DJ-1 possesses a reactive cysteine at position 106 (Cys-106), which undergoes preferential oxidation under oxidative stress. The critical role of this cysteine residue in the biological functioning of DJ-1 has been demonstrated^14^,^15^. Cys-106 in DJ-1 is gradually oxidized to cysteine sulfenic acid (Cys-SOH), cysteine sulfinic acid (Cys-SO_2_H), and cysteine sulfonic acid (Cys-SO_3_H). The acidic spot shift of DJ-1 observed by 2D-PAGE analysis of cells under oxidative stress arises from oxidation of the cysteine residue to either Cys-SO_2_H or Cys-SO_3_H. The former is chemically unstable and easily oxidized to the latter under normoxia; however, Cys-SO_2_H at position 106 of DJ-1 is stable because of the surrounding amino acid residues^16^. The Cys-SO_2_H form of DJ-1 is postulated to be the active form of DJ-1, based on studies that have shown a protective effect following a E18A point mutation, which depressed the pKa of Cys-106 and stabilized the Cys-SO_2_H form of Cys-106 in DJ-1^16^,^17^. Further oxidation of Cys-106 to Cys-SO_3_H leads to loss of biological function. DJ-1 thus acts as an oxidative stress sensor, detecting cellular redox status through the oxidation of Cys-106 and altering the activity of signal mediators and the expression levels of genes involved in anti-oxidative defence^1^,^3^,^18^.

PD is a progressive, age-related, neurodegenerative disorder, characterized by bradykinesia, rigidity and tremors^19^. These symptoms are caused by the degradation of dopamine neurons in the substantia nigra pars compacta of the midbrain and the subsequent depletion of striatal dopamine^20^. The pathological hallmark of PD is the presence of insoluble clumps of protein, called Lewy bodies, which contain α-synuclein^21^. Oxidative stress is a crucial mediator in the pathogenesis of PD. Increased levels of oxidation products, of lipids, proteins, and nuclear acids in nigral cells of PD patients, have been shown^22^,^23^. An increase in the amounts of oxidants such as copper and iron and a decrease in the amounts of anti-oxidants such as GSH and phospholipid peroxide GSH peroxidase (PH-GPx) have also been reported in the substantia nigra of PD patients^24^,^25^,^26^. The significance of DJ-1 in anti-oxidative defence and the loss of DJ-1 function in *PARK7* also indicate the role of oxidative stress in the pathogenesis of PD^1^,^2^,^3^,^4^.

The identification of a biomarker for PD in its early phase is vital for overcoming PD^27^. Current diagnosis of PD is dependent on recognizing the cardinal symptoms such as movement disorders; however, more than half of the dopamine neurons in the substantia nigra of the midbrain have been lost by the time the patient is diagnosed with PD^19^,^20^. The identification of a biomarker for PD at an early stage of the disease would serve not only to identify preclinical PD patients for preventive treatment but also facilitate the development of novel therapeutics for the prevention of the progression of PD. In this regard, there have been a number of attempts to develop *in vivo* imaging markers for dopamine neurons and iron levels in the substantia nigra^28^,^29^. The development of biochemical markers has also received much attention, and biomarkers related to oxidative stress, such as oxidized lipids and proteins, are leading candidates based on the pivotal role of oxidative stress in PD. Thus, oxidized DJ-1 could be a promising candidate as a biomarker for oxidative stress in PD^4^,^15^. Specific antibodies against Cys-106-oxidized DJ-1 (oxDJ-1), the enzyme-linked immunosorbent assay (ELISA) and immunostaining have been previously developed^30^,^31^. Immunohistochemical analysis suggests that, in the substantia nigra of midbrain, oxDJ-1 levels increase in the early phases of PD and then decrease at later stages of PD patients with dementia who have already lost almost all of their dopamine neurons^31^. Preliminary analysis in 15 PD patients also suggests that unmedicated PD patients have increased erythrocyte oxDJ-1 levels compared with those of both PD patients who have been treated with L-DOPA and/or dopamine agonists, and healthy subjects^30^. “Unmedicated PD patients” are those diagnosed with PD but not yet started on medications such as L-DOPA and/or dopamine agonists. Thus, the evidence suggests that DJ-1 oxidation in erythrocytes and in the brain occurs in PD patients, particularly during the early phases. DJ-1 has been shown to play a physiological role in the protection of erythrocytes from oxidative damage^32^. A change in the level of 4-hydroxy-2-nonenal (4-HNE)-modified DJ-1 in the whole blood of PD patients has also been reported, which indicates elevated levels of lipid peroxidation to form 4-HNE^33^. Furthermore, studies have reported increases in the levels α-synuclein oligomer/total protein, nitrite and protein carbonyls in erythrocytes of PD patients^34^,^35^,^36^. The elevation of oxDJ-1 levels in blood has been observed in animal models of PD that were induced by the administration of neurotoxins such as 1-methyl-4-phenyl-1,2,3,6-tetrahydropyridine (MPTP) and 6-hydroxydopamine (6-OHDA)^37^. Thus, the evidence suggests that DJ-1 oxidation in erythrocytes occurs in both PD patients and in animal models of PD.

In the present study, levels of oxDJ-1 in erythrocytes of PD patients (total number = 150) were determined, and the effects of PD medication on oxDJ-1 levels were quantified by using an oxidized DJ-1-specific antibody. Oxidized DJ-1 levels in the erythrocytes were also determined in a non-human primate model of PD induced by MPTP. Biochemical analysis of oxDJ-1 in erythrocytes suggests an interaction with 20S proteasome.

## Results

### Elevation of oxidized DJ-1 in the erythrocytes of PD patients in the early phase.

**Figure 1:**
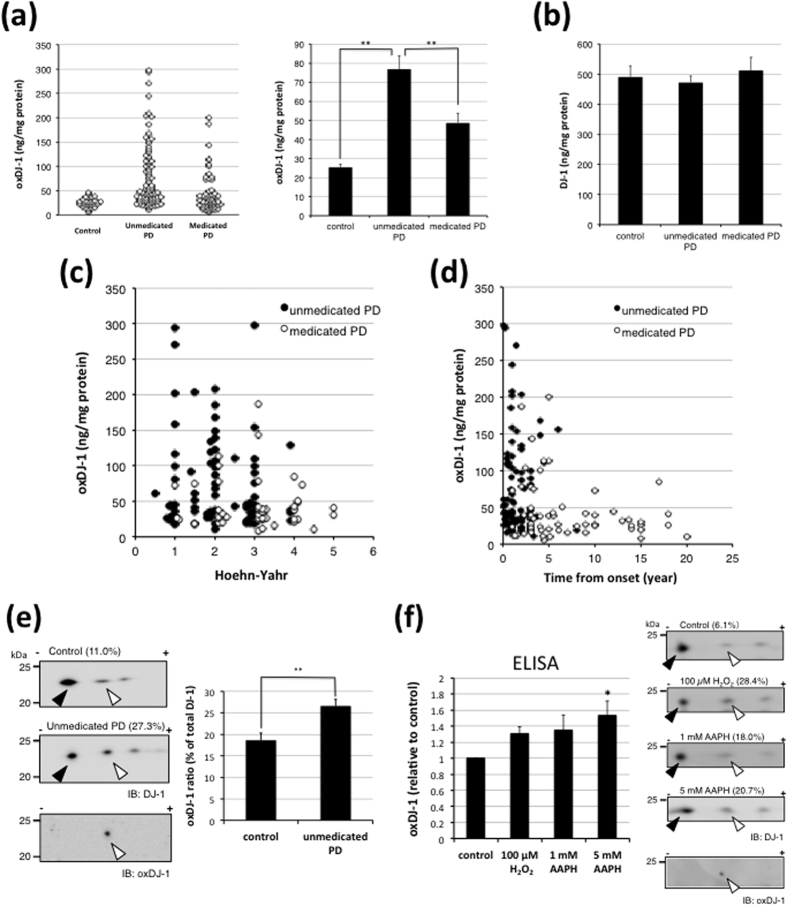
Elevation of oxDJ-1 levels in the erythrocytes of unmedicated PD patients. **(a–d)** Levels of oxDJ-1 (**a**) and DJ-1 (**b**) in erythrocytes of healthy controls (n = 33), unmedicated PD patients (n = 88) and medicated PD patients (n = 62). Individual data (**a**) and the means ± SEM (**a**,**b**) are shown. **P < 0.01, Steel-Dwass test, ANOVA. Individual oxDJ-1 levels in unmedicated (n = 82 for (**c**), n = 88 for (**d**) and medicated PD patients (n = 62) were plotted against Hoehn-Yahr stage (**c**) and time from onset in years (**d**). **(e)** Levels of oxDJ-1 in erythrocytes of healthy controls (n = 22) and unmedicated PD patients (n = 41) were evaluated by using 2D-PAGE and western blot for DJ-1 and oxDJ-1. The filled triangle and open triangle indicate native and oxidized DJ-1, respectively. The relative densities of oxDJ-1 normalized to total DJ-1 were estimated as described in the Methods; these values (%) are shown in parentheses for each analysis. In western blot for oxDJ-1, the result of healthy control is shown. Graphs display the relative densities of oxDJ-1 normalized to total DJ-1 (mean ± SEM). **P < 0.01, Student’s *t* test. **(f)** Whole blood of healthy control (20% in PBS, n = 4) was treated with oxidant, either H_2_O_2_ or AAPH, for 2 h, and then erythrocyte lysates were prepared. The lysates were then evaluated by a competitive ELISA for oxDJ-1 and 2D-PAGE and western blot for DJ-1. In ELISA, the relative means ± SEM are shown. *P < 0.05, Tukey-Kramer test, ANOVA. In 2D-PAGE, the relative mean densities of oxDJ-1 normalized to total DJ-1 are shown in parentheses. In western blot for oxDJ-1, the result of control condition is shown.

Levels of oxDJ-1 in erythrocytes from unmedicated PD patients (n = 8) were markedly higher than those of medicated PD patients and healthy subjects, as quantified by using a competitive ELISA system with an oxDJ-1-specific antibody^30^. In the present study, measurements obtained from unmedicated PD patients (n = 88) were significantly higher than those of both medicated PD patients and healthy subjects (Fig. 1a). PD was diagnosed on the basis of criteria reported by Calne *et al*.^38^ and classified into Hoehn-Yahr (H-Y) stages (1–5)^39^. Characteristics of PD patients, including type of medications, and healthy control subjects are summarized in Table 1. No statistically significant difference was observed in total DJ-1 levels, age, or H-Y stages between unmedicated PD patients and other groups (Fig. 1b and Table 1). Plotting the levels of oxidized DJ-1 against PD stage (H-Y 1–5) (Fig. 1c) showed that higher levels of oxDJ-1 in erythrocytes were present at early-stage PD, such as H-Y 1 and 2. Furthermore, when the oxDJ-1 levels were plotted against time from onset of PD, higher levels of oxDJ-1 were primarily observed within the first five years (Fig. 1d). Taken together, these results suggest that the levels of oxidized DJ-1 in erythrocyte increase during early-phase PD, particularly in unmedicated PD patients. The elevation of oxDJ-1 levels in erythrocytes was further analysed by using 2D-PAGE and western blotting techniques. The intensity of each spot was determined, and the ratio of the amount of oxDJ-1 to the total amount of DJ-1 was estimated. Although the sensitivity is relatively low, the identity of oxDJ-1 was confirmed by oxDJ-1-specific western blotting (Fig. 1e and Supplementary Fig. S1). A significant increase in the ratio of oxDJ-1 to DJ-1 in the erythrocytes of unmedicated PD patients was observed (Fig. 1e). The increase in the levels of oxDJ-1 in erythrocytes was further confirmed by using oxidant-treated whole blood. Whole blood was treated with either hydrogen peroxide or a water-soluble free-radical generator, 2,2′-azobis(2-amidinopropane) dihydrochloride (AAPH), then erythrocytes were collected and ruptured by addition of water to prepare lysates for analysis. The levels of oxDJ-1 in the lysates were determined by both ELISA and 2D-PAGE analysis, and the identity of oxDJ-1 in 2D-PAGE was confirmed by oxDJ-1-specific western blotting. The increase in oxDJ-1 levels induced by oxidative stress was observed by using both these methods (Fig. 1f).

### Elevation of oxidized DJ-1 levels in the erythrocytes of a non-human primate PD model induced by MPTP

**Figure 2:**
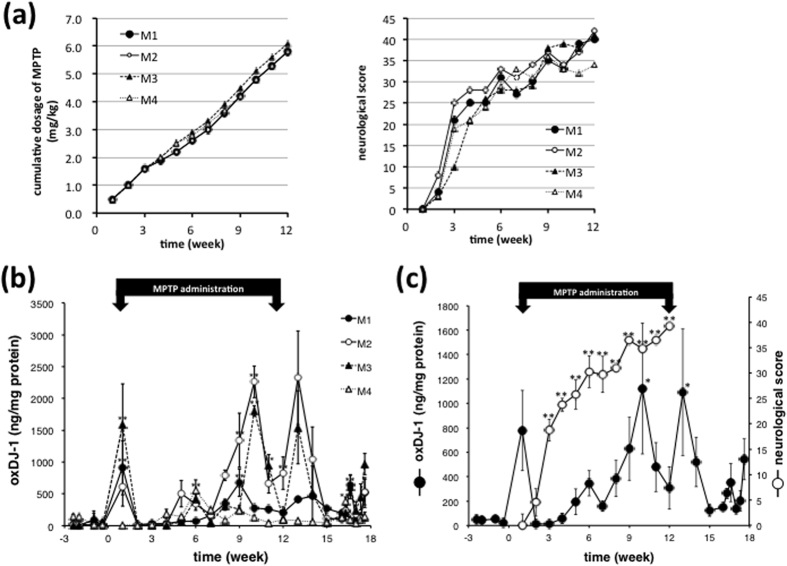
Elevation of oxidized DJ-1 levels in erythrocytes of MPTP-treated cynomolgus macaques. (**a)** Cumulative dose and neurological score of MPTP-treated cynomolgus macaques. Values from each macaque are shown. **(b,c)** During MPTP treatment, oxDJ-1 levels in erythrocytes from each monkey were determined by using competitive ELISA. The mean ± SEM of individual oxDJ-1 determinants (n = 4–6) are shown (**b**). The mean ± SEM of oxDJ-1 levels and neurological score (n = 4) are shown (**c**). *P < 0.05, **P < 0.01, Tukey-Kramer test, ANOVA, when compared with pretreatment (-1 week).

The elevation of oxDJ-1 levels in erythrocytes was further examined in a preclinical PD non-human primate model induced by MPTP^40^. The neurotoxin MPTP was administered to four monkeys through intramuscular (i.m.) injection either one or two times per week, and neurological scores were determined weekly over a period of 12 weeks^40^. Cumulative doses of MPTP and neurological scores are shown in Fig. 2a. Neurological scores rapidly increased three weeks after the first administration of MPTP and scores increased until the final dose (Fig. 2a). The behavioural data suggest degeneration of dopamine neurons three weeks after the first administration of MPTP. Oxidized DJ-1 levels in erythrocytes were determined by ELISA. A transient change in oxDJ-1 levels in erythrocytes was suggested after the first week of treatment, which appears to be an acute phase response (Fig. 2b). After this change, significant increases in oxDJ-1 levels were observed at some points (Fig. 2b). Individual neurological scores and oxDJ-1 levels are summarized in Fig. 2c. In the non-human primate model of PD, oxDJ-1 levels varied between individuals, with high and low responders, even though neurological scores between individuals were similar.

### Dimeric and higher polymer forms of oxidized DJ-1 in the erythrocytes of unmedicated PD patients

**Figure 3:**
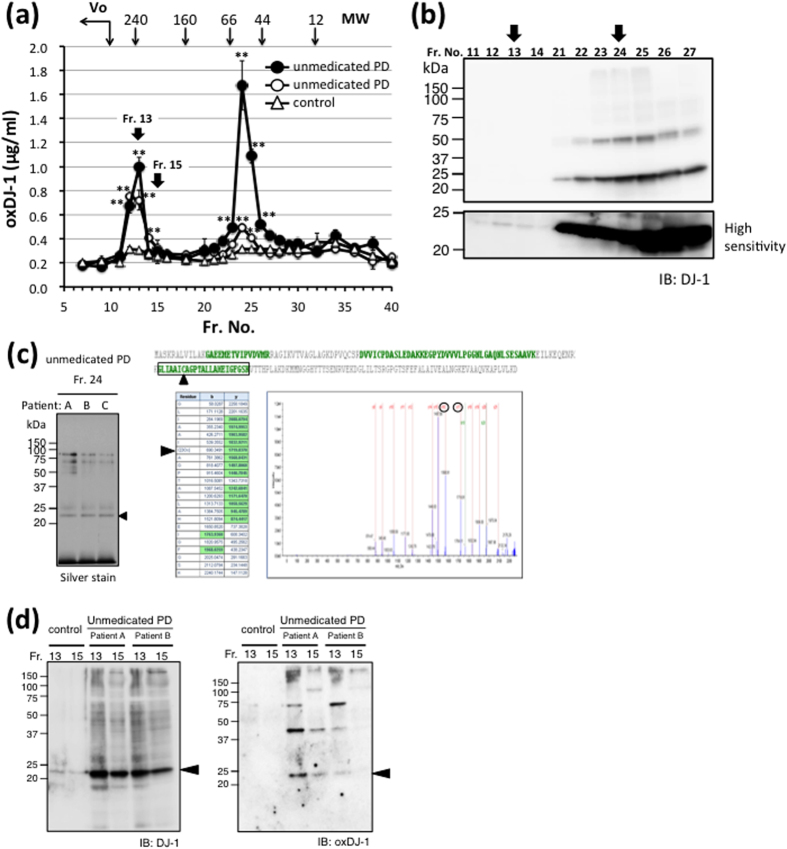
Biochemical properties of oxidized DJ-1 detected in the erythrocytes of unmedicated PD patients. (**a,b)** The erythrocyte lysates of unmedicated PD patients and healthy controls (60 mg protein) were separated by gel chromatography, and the oxDJ-1 content of each fraction was determined by using competitive ELISA (**a**). The mean ± SD (n = 3) is shown. **P < 0.01, Tukey-Kramer test, ANOVA, when compared with control buffer. The following proteins were used to determine molecular weight: 240 K, mouse catalase; 160 K, bovine γ-globulins; 66 K, bovine serum albumin; 44 K, ovalbumin; 12 K, horse cytochrome *c*. The void volume (Vo) of this chromatography is indicated. Each fraction was also subjected to western blot analysis for DJ-1. Typical result of western blotting is shown. Major oxDJ-1 peak (Fr. 13 and 24) indicated by black arrows (**b**). **(c)** Oxidized DJ-1 in Fr. 24 was immunoprecipitated by using an antibody against oxDJ-1, and immunoprecipitants were visualized by silver staining. The band corresponding to oxDJ-1 was subjected to in-gel digestion and MALDI-TOF MS analysis. Identified peptide sequences with confidence intervals greater than 95% are shown in green. The MS/MS spectra of peptides predicted to contain Cys-106-SO_3_H. The MS/MS spectra provide sequence data for unequivocal assignment of oxDJ-1 (100-122), the Cys that is oxidized to Cys-SO_3_H (arrowhead). **(d)** Proteins in Fr. 13 and 15 of unmedicated PD patients and healthy controls were subjected to western blot analysis for DJ-1 and oxDJ-1. Typical result of western blotting is shown.

The biochemical properties of oxDJ-1 detected in erythrocytes of early stage PD patients were further investigated. The proteins in erythrocyte lysates were separated by gel chromatography and the oxDJ-1 content of each fraction was determined by ELISA. Two oxDJ-1 peaks were observed, one at 45 kDa (fraction number 24, Fr. 24) and a second at more than 200 kDa (Fr. 13) (Fig. 3a). These two peaks were observed in all samples with high oxDJ-1 levels (n = 6, data not shown). To evaluate the contamination of unfolded and unsoluble oxDJ-1 in hemolysate samples, it was confirmed that the ultracentrifugation at 105,000 × g for 1 h did not influence on the oxDJ-1 determinants (data not shown). Western blotting indicated the presence of DJ-1 in both fractions, and a 45 kDa band, which might be the dimeric form of DJ-1, was also observed in Fr. 24 (Fig. 3b). In the case of Fr. 24, which corresponds to the molecular weight of the dimeric form of oxDJ-1, immunoprecipitation with an oxDJ-1-specific antibody was conducted and the proteins in the precipitants were visualized by silver staining (Fig. 3c). The stained band was excised, trypsinized and subjected to LC-MS/MS analysis, which confirmed the oxidation of Cys-106 to Cys-SO_3_H (Fig. 3c). In the case of Fr. 13, which corresponds to the molecular weight of higher polymeric forms, no bands were obtained upon immunoprecipitation with either oxDJ-1 or DJ-1 antibodies. Western blotting confirmed the presence of both DJ-1 and oxDJ-1 in the higher polymer fraction of unmedicated PD patients (Fig. 3d). Both DJ-1 and oxDJ-1 was not detected in the higher polymer fraction of healthy control subjects (Fig. 3d). Western blot analysis of Fr. 13 and Fr. 15 suggested higher levels of oxDJ-1 and DJ-1 in Fr. 13 than in Fr. 15 (Fig. 3d), which is consistent with the results obtained by using ELISA (Fig. 3a).

### Interaction of oxidized DJ-1 with 20S proteasome in erythrocytes of unmedicated PD patients.

**Figure 4:**
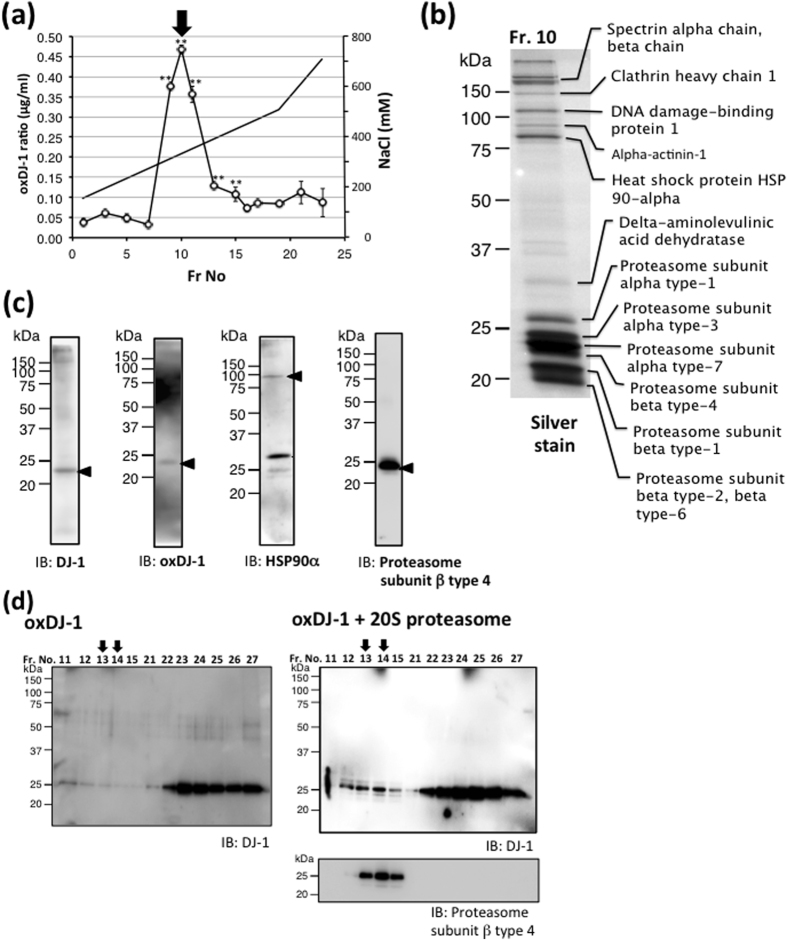
Biochemical properties of the polymer form of oxidized DJ-1 detected in the erythrocytes of unmedicated PD patients. **(a)** Proteins containing the polymer form of oxDJ-1 were separated by DEAE column chromatography, and the oxDJ-1 content of each fraction was determined by using competitive ELISA. The mean ± SD (n = 3) was shown. **P < 0.01, Tukey-Kramer test, ANOVA, when compared with buffer control. A major oxDJ-1 peak (Fr. 10) is indicated by the black arrow. **(b)** Proteins eluted in Fr. 10 by DEAE column chromatography were visualized by silver staining, and each band was subjected to in-gel digestion and MALDI-TOF MS analysis. Identified proteins are indicated. **(c)** Proteins eluted in Fr. 10 by DEAE column chromatography were subjected to western blot analysis for DJ-1, oxDJ-1, HSP90α, and proteasome subunit β type 4. **(d)** 20 S proteasome purified from human erythrocytes was incubated for 18 h at 4 °C in either the presence or absence of purified recombinant human oxDJ-1 in 50 mM HEPES buffer (pH 7.4) supplemented with 10% glycerol, 2 mM ATP and 2 mM DTT. Samples were then separated by gel chromatography, and each fraction was subjected to western blot analysis.

The high polymer form of oxDJ-1 obtained by gel chromatography was further separated by anion-exchange chromatography. The fraction containing oxDJ-1 was applied to DEAE Sepharose, and gradually eluted with NaCl solution. Oxidized DJ-1 immunoreactivity was observed as a single peak around Fr. 10 (Fig. 4a). The proteins contained in Fr. 10 were separated by SDS-PAGE and visualized by silver stain. The stained band was excised, trypsinized, and subjected to LC-MS/MS analysis. Subunits of 20S proteasome and HSP90, which have been reported to interact with DJ-1^13^,^41^, were detected by LC-MS/MS analysis (Fig. 4b), whereas none of the 19S subunits of 26S proteasome were observed. Proteins such as DJ-1, oxDJ-1, HSP90, and proteasome subunit type 4 were contained in Fr.10, as demonstrated by western blotting (Fig. 4c). To determine a potential interaction between these proteins, 20S proteasome purified from human erythrocytes and recombinant oxidized DJ-1 were incubated at 4 °C in the presence of ATP and dithiothreitol (DTT), as previously described^13^. Samples were then separated by gel chromatography and subjected to western blotting. Oxidized DJ-1 levels in the higher molecular weight fraction increased, suggesting an interaction between oxDJ-1 and 20 S proteasome (Fig. 4d). In the presence of 20S proteasome, 16% of total oxDJ-1 was detected in the higher molecular weight fraction. The addition of human HSP90α recombinant protein did not alter the interaction between oxDJ-1 and 20S proteasome (Supplementary Fig. S2). Taken together, these results suggest an interaction of oxDJ-1 with 20S proteasome in the erythrocytes of unmedicated PD patients. To further establish whether native DJ-1 binds to 20S proteasome, 20S proteasome and recombinant DJ-1 were incubated at 4 °C and then samples were subjected to gel chromatography. It was found that DJ-1 levels in the higher molecular weight fraction increased, and 25% of total DJ-1 was detected in the higher molecular weight fraction in the presence of 20S proteasome (Supplementary Fig. S3). In accordance with a previous report^13^, it is demonstrated that oxidation of DJ-1 does not enhance the DJ-1/20S proteasome association.

## Discussion

DJ-1, the product of the causative gene of a familial form of PD, undergoes preferential oxidation of Cys-106 under oxidative stress, and this reactive Cys is necessary for the anti-oxidative function of DJ-1. Oxidative stress plays a significant role in the onset as well as the progression of PD. DJ-1 acts as a sensor for oxidative stress. Thus, levels of oxDJ-1 could be an early indicator of oxidative stress related to PD. The present study demonstrates significant elevation of oxDJ-1 levels in the erythrocytes of unmedicated PD patients and an interaction between oxDJ-1 with 20S proteasome. The unoxidized form of DJ-1 recombinant protein clearly interacted with 20S proteasome in the present study, and oxidation of DJ-1 does not enhance the DJ-1/20S proteasome association, as reported previously^13^. At present, the precise molecular mechanisms of interaction between oxDJ-1/DJ-1 and 20S proteasome have not been elucidated, and further studies are necessary to understand this interaction. Furthermore, several studies have reported that post-translationally modified proteins such as α-synuclein oligomer and protein carbonyls accumulate in the erythrocytes of PD patients^33^,^34^,^35^,^36^. These results together with the current findings suggest that oxDJ-1 interacts with the 20S proteasome, inhibits its activity, and is related to the accumulation of modified proteins in the erythrocytes of PD patients.

High levels of oxDJ-1 are observed in unmedicated PD patients with low H-Y values and within five years of the onset of PD. The current study confirmed the presence of increased levels of oxDJ-1 in the erythrocytes of unmedicated PD patients. Oxidation of DJ-1 is induced by several oxidants and neurotoxins, and the decrease in cellular GSH levels could enhance DJ-1 oxidation^14^,^42^,^43^. The present study shows that DJ-1 was oxidized following treatment of whole blood with either hydrogen peroxide or free radicals. Levels of reduced and total GSH in the erythrocytes of unmedicated PD patients did not change significantly in the current study (Supplementary Fig. S4). DJ-1 has been reported to play a role in protection of the erythrocytes from oxidative damage^32^, and the present data indicate the accumulation of oxDJ-1, formed through irreversible oxidation of Cys106, in the erythrocytes. The pKa of reactive Cys-106 has been reported to be depressed to 5.4^16^, which is lower than that of GSH thiol group, 8.8. The concentrations of DJ-1 and reduced GSH in human erythrocytes were calculated as 23 pmol/mg protein and 6 nmol/mg protein, respectively (Table 1 and Supplementary Fig. S4). Reaction of DJ-1 with H_2_O_2_ is not enzymatic, while cellular GSH is newly synthesized and oxidized GSH will be reduced by GSH reductase. The difference between cultured cells and PD patients might be caused by chronic exposure to oxidative stress in PD patients. Levels of oxDJ-1 *in vivo* are determined by a balance between rates of formation and degradation, by secondary reactions and by its stability *in vivo*. Further investigations are needed to elucidate the precise molecular mechanism of DJ-1 oxidation.

The present study defined “medicated PD patients” as those treated with PD medications for more than six months. A possible beneficial effect of PD medication is a reduction of oxidative stress in peripheral tissues, as demonstrated by reduced oxDJ-1 levels in blood. Changes in peripheral tissues in PD patients have been demonstrated previously. Peripheral nervous system disorders have also been used for the diagnosis of PD. Post-mortem cardiac samples showed decreased levels of tyrosine hydroxylase-positive axons, which is a marker for sympathetic axons, indicating degeneration of cardiac nerves in PD patients^44^,^45^. *Meta*-iodobenzylguanidine (MIBG), an analogue of noradrenaline, is actively taken up and stored in sympathetic nerves, and cardiac uptake of radiolabelled MIBG has been used for the diagnosis of PD^46^. An association between oxDJ-1 levels in erythrocytes and cardiac uptake of MIBG has also been reported^47^. Thus, changes in peripheral tissues, such as erythrocytes and the heart, in patients with PD could be utilized as biomarkers for the diagnosis of early-phase PD.

In accordance with the clinical findings, elevated levels of erythrocyte oxDJ-1 were observed in a non-human primate model of PD. Oxidized DJ-1 levels transiently increased one week after the first administration of MPTP, while there was no sign of neurological deficits. After the initial change of oxDJ-1 levels, these levels also increased during subsequent administration of MPTP, suggesting an acute and chronic response to MPTP; the latter response might be related to the degeneration of dopaminergic neurons. An increase of erythrocyte oxDJ-1 levels was also observed in a mouse model of PD, following a single administration of MPTP^37^. In mice, levels of brain dopamine decreased significantly three days after MPTP treatment, whereas a significant elevation of oxDJ-1 was observed four weeks after MPTP treatment. In 6-OHDA-treated rats, increased levels of erythrocyte oxDJ-1 expression were observed two weeks after a single treatment of 6-OHDA^37^. The data obtained from rodent models suggest the emergence of a chronic response of oxDJ-1 to brain dopamine depletion that appears long after administration of the neurotoxin.

An immunohistochemical study showed accumulation of oxDJ-1 in the substantia nigra of PD patients, particularly during the early phases^31^. In that study, oxDJ-1 levels were analysed in human brain sections, which were classified according to the extent of Lewy body (LB) formation. The extent of LB formation comprises: (1) LB stage 0: no LBs; (2) LB stage I: scattered LBs without cell loss; (3) LB stage II: abundant LBs with macroscopic loss of pigmentation in the substantia nigra in the areas containing LBs but without attributable parkinsonism or dementia; and (4) LB stage III-PD: PD without dementia^31^,^48^. Oxidized DJ-1 immunoreactivity in the substantia nigra was observed at LB 0, and maximum levels were observed for LB stage II and III-PD cases. These findings indicate that oxDJ-1 appears in the brain before the emergence of PD symptoms^31^. Thus, the generation of oxDJ-1 in the substantia nigra as well as in erythrocytes is an event that occurs during early-stage PD.

In conclusion, the present study clearly shows the generation of oxDJ-1 in erythrocytes in early stage, unmedicated PD patients and in non-human primates with PD symptoms. In addition, oxDJ-1 interacts with 20S proteasome in the erythrocytes of PD patients. The current findings suggest that oxidative stress is systemically present in PD patients and that anti-oxidative therapy could be beneficial for treatment of PD. The current observations also suggest that an early pathophysiological mechanism operates in PD; this finding could be used to develop methods for the efficient and early diagnosis of PD.

## Methods

### Chemicals

Hydrogen peroxide (H_2_O_2_) and isopropyl-β-d-1-thiogalactopyranoside (IPTG) were purchased from Wako Pure Chemical Industries, Osaka, Japan; 3,3′,5,5′-tetramethylbenzidine (TMB), 1-methyl-4-phenyl-1,2,3,6-tetrahydropyridine hydrochloride (MPTP), and anti-β actin (AC-15) were obtained from Sigma-Aldrich, St. Louis, MO; nickel-nitrilotriacetic acid (Ni-NTA) agarose was obtained from Qiagen GmbH, Hilden, Germany; and protease inhibitor cocktail tablets were obtained from Nacalai Tesque, Kyoto, Japan. Monoclonal antibodies (mAbs) against oxDJ-1 were prepared as previously described^30^,^31^. In each experimental system for the analysis of oxDJ-1, the most suitable mAb clone was selected based on the specificity and sensitivity. Other chemicals used were of the highest quality commercially available.

### Subjects

All experiments were performed in accordance with relevant guidelines and regulations. All procedures were approved by the Ethics Committees of Doshisha University (approval number 1109) and the medical institutions of Sapporo Medical University and Kinki University. Informed consent was obtained from all subjects. PD was diagnosed on the basis of the criteria reported by Calne *et al*.^35^ and classified into five stages (H-Y 1–5)^39^. Information on PD patients and healthy control subjects is summarized in Table 1. Unmedicated PD patients [n = 88; male/female: 46/42; age: 69.2 ± 9.6 (years ± S.D.), age range: 35–86 years], medicated PD patients [n = 62; male/female: 28/34; age: 67.3 ± 11.3 (years ± S.D.), age range: 37–83 years], and healthy controls [n = 33; male/female: 15/18; age: 62.8 ± 7.7 (years ± S.D.), age range: 50–73 years] were recruited for this study (Table 1). The term “unmedicated PD patients” in this study refers to patients diagnosed with PD but not yet started on PD treatment, such as L-DOPA and/or dopamine agonists. The term “medicated PD patients” in this study refers to patients treated with PD medications, such as L-DOPA and/or dopamine agonists, for more than six months. It has previously been reported that levels of oxDJ-1 in erythrocytes of unmedicated PD patients (n = 8) are higher than those of medicated PD patients (n = 7) and healthy controls (n = 18)^30^. These values have been included in the present study.

Blood samples were collected in tubes containing ethylenediamine tetraacetic acid. Plasma was collected following centrifugation of the blood sample at 1,580 × g for 10 min at 4 °C, and the erythrocytes were washed twice with a fourfold volume of saline. After washing, erythrocytes were treated with a fivefold volume of Milli-Q water. Hemolysate samples were centrifuged at 17,000 × g for 15 min at 4 °C, and the supernatants were used for ELISA. Protein content was determined by using a BCA protein assay kit (Pierce, Rockford, IL) with bovine serum albumin as the standard.

### Measurement of oxidized DJ-1 and total DJ-1

The Preparation of Cys-106-oxidized DJ-1 recombinant protein for ELISA was previously described^30^,^31^. Full-length human DJ-1 cDNA (570 bp, NM_007262) was cloned into pEXP1-DEST and transformed into *Escherichia coli* strain BL21(DE3)pLysS, and a fusion protein was obtained with a 6-His tag at the amino terminus. Protein expression was induced by the addition of 0.5 mM IPTG. After 2 h, DJ-1 in the cells was oxidized by treatment with 50 mM H_2_O_2_ for 15 min at 37 °C. The oxidized DJ-1 recombinant protein was purified by using Ni-NTA agarose. The oxidation of Cys-106 was confirmed by matrix-assisted laser desorption/ionization time-of-flight mass spectrometry (MALDI-TOF MS), described below. We confirmed by mass spectrometry analysis that Cys106 of recombinant oxDJ-1 protein was fully oxidized to sulfonic acid, and it was also confirmed that the unoxidized form of Cys106 was not detected in oxDJ-1 recombinant protein. Preparation of horseradish peroxidase (HRP)-conjugated oxidized DJ-1 recombinant protein was conducted according to Nakane *et al*.^49^. Monoclonal antibodies against oxDJ-1 were prepared as previously described^30^,^31^.

Quantitation of Cys-106-oxidized DJ-1 was determined by competitive ELISA, as previously described^30^. Briefly, 96-well microtiter plates were coated with mouse anti-Cys-106-oxidized DJ-1 mAb dissolved in 0.05 M sodium bicarbonate buffer (pH 9.6) for 18 h at 4 °C. The wells were washed four times with phosphate-buffered saline (PBS) containing 0.05% Tween 20 (PBS-Tween), and incubated with PBS containing 0.1% bovine serum albumin for 1 h at 37 °C. The wells were again washed four times, and either standard Cys-106-oxidized DJ-1 or the clinical sample (diluted in PBS-Tween containing 0.1% bovine serum albumin) and HRP-conjugated Cys-106-oxidized DJ-1 were added to each well and incubated for 2 h at 37 °C. Finally, the plates were washed eight times and air-dried. TMB was added to each well, and the enzyme-substrate reaction was allowed to proceed for 30 min in the dark. The reaction was stopped by addition of 1 M sulfuric acid to each well. Absorbance was measured at 450 nm with an OPTImax plate reader (Molecular Devices). The content of Cys-106-oxidized DJ-1 in each well was calculated by using the absorbance values of the standard protein. Two different mAbs for oxDJ-1, clones 7411 and 3805, were used for ELISA; both showed similar levels of antibody sensitivity to the antigen.

DJ-1 content was measured with a CircuLex Human DJ-1/PARK7 ELISA kit (CY-9050, CircuLex, Japan) according to the supplier’s protocol^50^.

### SDS-PAGE and 2D-PAGE for western blotting

For western blot analysis, cell lysates from each sample were reduced and denatured in 63 mmol/L Tris-HCl (pH 6.8) containing 1% mercaptoethanol, 2% SDS, 5% sucrose, and 0.012% bromophenol blue for 5 min at 95 °C. The reduced protein mixture was then separated on a 12.5% SDS-PAGE gel. For the first dimension of 2D-PAGE, immobilized pH gradient gel strips (pH 4-7; non-linear, 7 and 13 cm, GE Healthcare Bio-Science, Uppsala, Sweden) were used. The samples were mixed with sample buffer [9 M urea, 5% 3-[(3-cholamidopropyl)dimethylammonio]propanesulfonate, 65 mM dithioerythritol (DTE), and 0.5% ampholyte (pH 4–7)], applied on a gel, and rehydrated for 18 h. The electrophoresis voltage was increased stepwise to either 5,000 or 8,000 V at a maximum current of 200 mA for 3–5 h. Each strip was equilibrated in 50 mM Tris-HCl (pH 8.8) containing 6 M urea, 2% SDS, 30% glycerol, and 20 mM DTE for 20 min, and then separated on a 12.5% SDS-PAGE gel.

After separation by either SDS-PAGE or 2D-PAGE, the samples were transferred to an Immobilon-P Transfer Membrane (Millipore, Bedford, MA) for western blot analysis. The membranes were blocked in 5% skimmed milk powder (Snow Brand Milk Products, Tokyo, Japan) dissolved in Tris-buffered saline (pH 7.4) containing 0.1% Tween 20 (TBS-T), and incubated with mouse anti-DJ-1 mAb (clone 3E8, 1 μg/ml, Medical & Biological Laboratories, Nagoya, Japan), anti-oxDJ-1 mAb (clone M106^31^, 1 μg/ml), anti-HSP90α polyclonal Ab (ADI-SPS-771, 1,000-dilution, Enzo Life Sciences, Farmingdale, NY), or anti-Proteasome subunit type 4 polyclonal Ab (ab106816, 0.1 μg/ml, Abcam, Cambridge, MA) at 4 °C for 18 h, washed with TBS-T, incubated with HRP-conjugated secondary antibodies for at least 1 h, and washed with TBS-T. The immunoreactivity was visualized with Immobilon Western (Millipore) and an LAS-4000 luminescence imager (Fujifilm, Tokyo, Japan). The relative densities were determined with the MultiGauge software (Fujifilm). The ratio of oxDJ-1 was calculated from the equation: ratio of oxDJ-1 = (intensity of oxDJ-1)/(intensity of all three DJ-1 spots). For silver staining, the separated proteins were stained with the Dodeca Silver Stain Kit (Bio-Rad Laboratories, Hercules, CA).

### Animal experiments

All animal experiments described in this study fully conformed to the guidelines outlined in the Guide for the Care and Use of Laboratory Animals of Japan and were approved by the Animal Care Committee of Doshisha University (approval no. 1230) and Hamamatsu Pharma Research, Inc (approval no. HPRIRB-36). MPTP (0.1–0.5 mg/kg, i.m.) was administered to four male *Macaca fascicularis* (cynomolgus) monkeys either one or two times per week until symptoms of parkinsonianism were observed. Parkinsonian symptoms were quantified by using a rating scale, as previously reported^40^.

### Fractionation of erythrocyte proteins by gel chromatography and ion exchange chromatography

AKTAprime plus (GE Healthcare) was used for the fractionation of erythrocyte proteins. In the case of gel filtration chromatography, the erythrocyte lysate was fractionated by using HiPrep 16/60 Sephacryl Columns S-300 HR (GE Healthcare) equilibrated with 20 mM Tris–HCl (pH 7.4) containing 150 mM NaCl. For ion exchange chromatography, oxDJ-1 immunoreactive fractions were applied to a HiTrap DEAE FF (GE Healthcare) equilibrated with 20 mM Tris–HCl (pH 7.4) containing 150 mM NaCl. Proteins were eluted with a NaCl gradient (150–700 mM).

### Immunoprecipitation

A monoclonal antibody against oxidized DJ-1 (clone 3805) was used for the immunoprecipitation experiment. The antibody was coupled to protein A Sepharose CL-4B (GE Healthcare) with dimethyl pimelimidate dihydrochloride. The anti-oxDJ-1 antibody-conjugated Sepharose CL-4B was applied to the erythrocyte fraction and incubated for 18 h at 4 °C. The column was washed with PBS and eluted with PBS containing 0.8% SDS, 0.004% bromophenol blue, 0.004% malachite green and 4% glycerol. Samples were subjected to SDS-PAGE in slab gels (12.5% gel) under reducing conditions, as described above.

### LC-MS/MS analysis

The identification of protein and the oxidation of Cys-106 in oxidized DJ-1 were confirmed by MALDI-TOF MS analysis, as described previously^31^. Briefly, proteins were separated by SDS-PAGE and visualized by silver staining. The stained band was excised and treated with sequence-grade modified trypsin (Promega). In-gel digestion was performed for 18 h at 37 °C. The resulting peptides were extracted and concentrated with a SpeedVac (Thermo Electron). The samples were mixed with α-cyano-4-hydroxycinnamic acid and subjected to MALDI-TOF MS analysis (4800 Plus; AB SCIEX, Framingham, MA). For detailed sequence analysis, peptide samples were separated and spotted on a plate for MALDI-TOF MS analysis with a capillary liquid chromatography (LC) system coupled to an auto-spotter (DiNa ASM-T-MaP; KYA TECH, Tokyo, Japan). Data were processed with the ProteinPilot 3.0 software (AB SCIEX). UniProtKB/Swiss-Prot protein databases were used to identify peptide fragments.

### Interaction analysis

Oxidized DJ-1 recombinant protein (30 pmol) was preincubated with purified human 20S proteasome (BML-PW8720-0050, Enzo Life Sciences) at a 1:1 molar ratio for 18 h, in 50 μL, 50 mM HEPES buffer (pH 7.4) supplemented with 10% glycerol, 2 mM ATP and 2 mM DTT, at 4 °C. Samples were diluted to 500 μL with 20 mM Tris–HCl (pH 7.4) containing 150 mM NaCl, and then immediately submitted to gel filtration chromatography as described above. In the case of HSP90α addition, 30 pmol of human HSP90α recombinant protein (SPR-101A, StressMarq Biosciences) was added to the reaction mixture.

### Statistical analysis

The difference between determinations was statistically analysed with either Student’s *t* test or analysis of variance (ANOVA) using Tukey-Kramer test for multiple comparisons. In the case of non-parametric data in human samples, Steel-Dwass test was conducted for ANOVA. Kruskal-Wallis test was conducted before Steel-Dwass test. Values of *P* < 0.05 were considered as significant.

## Additional Information

**How to cite this article**: Saito, Y. *et al*. Oxidation and interaction of DJ-1 with 20S proteasome in the erythrocytes of early stage Parkinson’s disease patients. *Sci. Rep.* **6**, 30793; doi: 10.1038/srep30793 (2016).

## Supplementary Material

Supplementary Information

## Figures and Tables

**Table 1:** Summary of research participants.

	Unmedicated PD	Medicated PD	Healthy control
	(n = 88)	(n = 62)	(n = 33)
Age in years^a^	69.2 ± 9.6	67.3 ± 11.3	62.8 ± 7.7
Male/female	46/42	28/34	15/18
Hoehn-Yahr^a^	2.1 ± 0.9	2.7 ± 1.1	—
Medications	—	62	—
L-dopa		58	
Agonists		47	
L-dopa + agonists		43	
Oxidized DJ-1 (ng/mg protein)^a^	77 ± 66 **	49 ± 45	25 ± 9
DJ-1 (ng/mg protein)^a^	473 ± 146	512 ± 199	491 ± 166

^a^The mean values are shown with standard deviation. **P < 0.01 (Steel-Dwass, ANOVA) compared with medicated PD and healthy control. Significant difference between groups was only observed in oxidized DJ-1.
