# Supplementary material for: Oxidation and interaction of DJ-1 with 20S proteasome in the erythrocytes of early stage Parkinson’s disease patients
Source: Sci Rep. 2016 Jul 29;6:30793. doi: 10.1038/srep30793 (PMC4965792; doi:10.1038/srep30793)
Supplement: Supplementary Information [file srep30793-s1.pdf]

Saito et al.,
Supplementary Figure

Title: Oxidation and interaction of DJ-1 with 20S proteasome in the erythrocytes of early stage Parkinson's disease patients

Authors: Yoshiro Saito[1,2], Yoko Akazawa-Ogawa[2], Akihiro Matsumura[3], Kazumasa Saigoh[4], Sayoko Itoh[5], Kenta Sutou[1], Mayuka Kobayashi[1], Yuichiro Mita[1], Mototada Shichiri[2], Shin Hisahara[3], Yasuo Hara[6], Harutoshi Fujimura[7], Hiroyuki Takamatsu[5], Yoshihisa Hagihara[2], Yasukazu Yoshida[2], Takao Hamakubo[8], Susumu Kusunoki[4], Shun Shimohama[3], and Noriko Noguchi[1]

Affiliations: [1] Systems Life Sciences laboratory, Department of Medical Life Systems, Faculty of Life and Medical Sciences, Doshisha University, Kyotanabe, Kyoto 610-0394, Japan. [2] National Institute of Advanced Industrial Science and Technology (AIST), Ikeda, Osaka 563-8577, Japan. [3] Department of Neurology, School of Medicine, Sapporo Medical University, Sapporo 060-8556, Japan. [4] Department of Neurology, Kinki University Faculty of Medicine, Osaka 589-8511, Japan. [5] Hamamatsu Pharma Research Inc., Hamamatsu 431-2103, Japan. [6] Hara Clinic, Ikeda, Osaka 563-0025, Japan. [7] Department of Neurology, National Hospital Organization Toneyama National Hospital, Toyonaka, Osaka 560-8552, Japan. [8] Laboratory of Systems Biology and Medicine, Research Center for Advanced Science and Technology, University of Tokyo, Tokyo 153-0041, Japan. Correspondence and requests for materials should be addressed to Y.S. (email: ysaito@mail.doshisha.ac.jp) and N.N. (email: nnoguchi@mail.doshisha.ac.jp).

S1

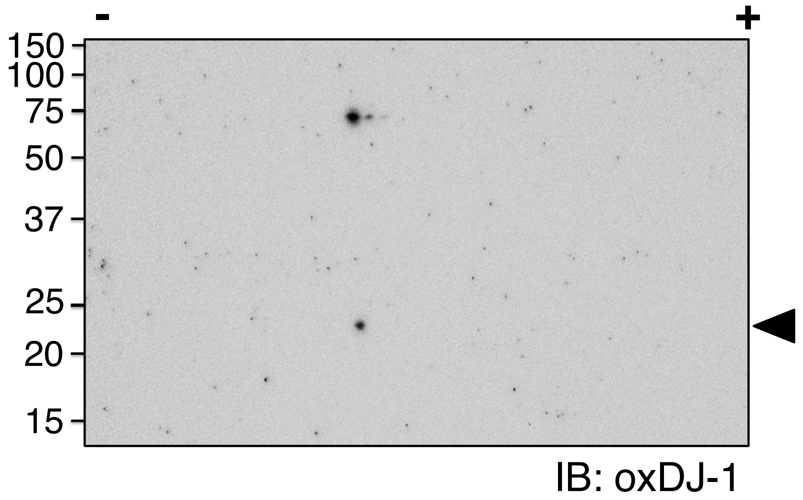

Supplementary Figure S1 │ Oxidized DJ-1 in erythrocytes of healthy controls was confirmed by using 2D-PAGE and western blot for oxDJ-1.

S2

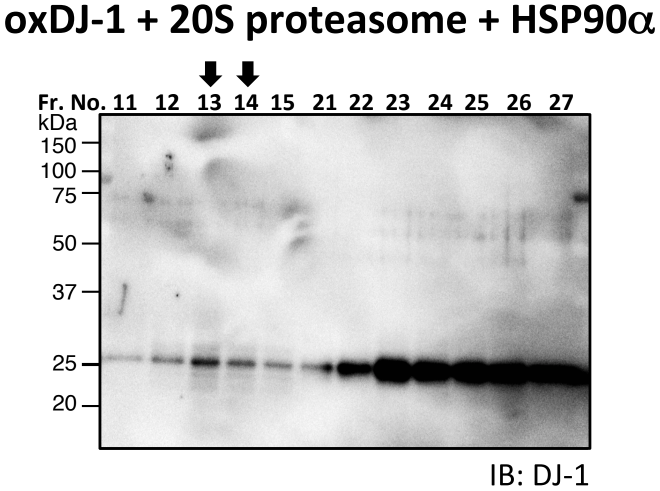

Supplementary Figure S2 │ Effects of recombinant HSP90a on the interaction between oxDJ-1 and 20S proteasome. Oxidized DJ-1 and 20S proteasome were incubated in the presence of recombinant HSP90a, and then subjected to gel chromatography.

Title: Oxidation and interaction of DJ-1 with 20S proteasome in the erythrocytes of early stage Parkinson's disease patients

Authors: Yoshiro Saito[1,2], Yoko Akazawa-Ogawa[2], Akihiro Matsumura[3], Kazumasa Saigoh[4], Sayoko Itoh[5], Kenta Sutou[1], Mayuka Kobayashi[1], Yuichiro Mita[1], Mototada Shichiri[2], Shin Hisahara[3], Yasuo Hara[6], Harutoshi Fujimura[7], Hiroyuki Takamatsu[5], Yoshihisa Hagihara[2], Yasukazu Yoshida[2], Takao Hamakubo[8], Susumu Kusunoki[4], Shun Shimohama[3], and Noriko Noguchi[1]

S3

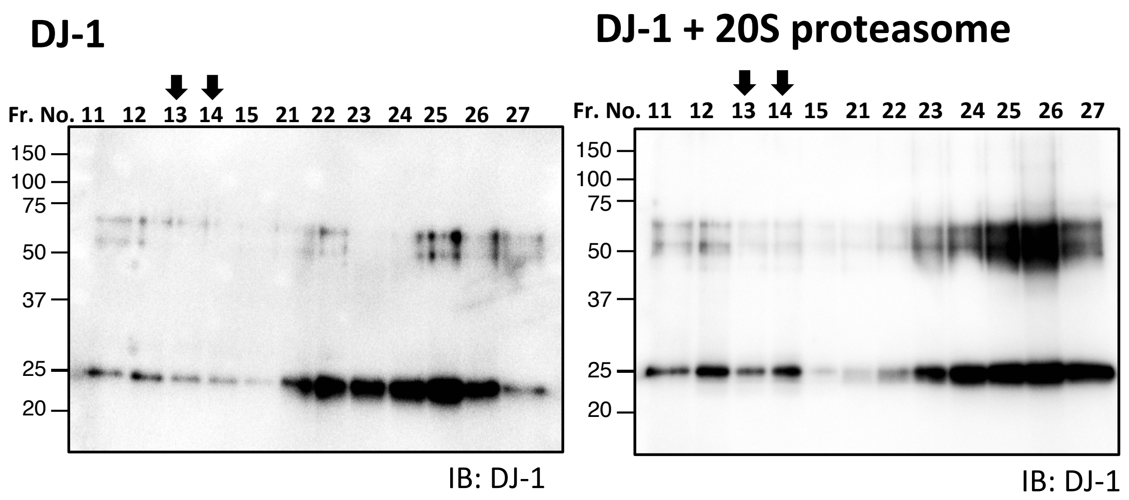

Supplementary Figure S3 | The interaction between DJ-1 and 20S proteasome. Native DJ-1 and 20S proteasome were incubated for 18 h at 4°C, and then subjected to gel chromatography.

S4

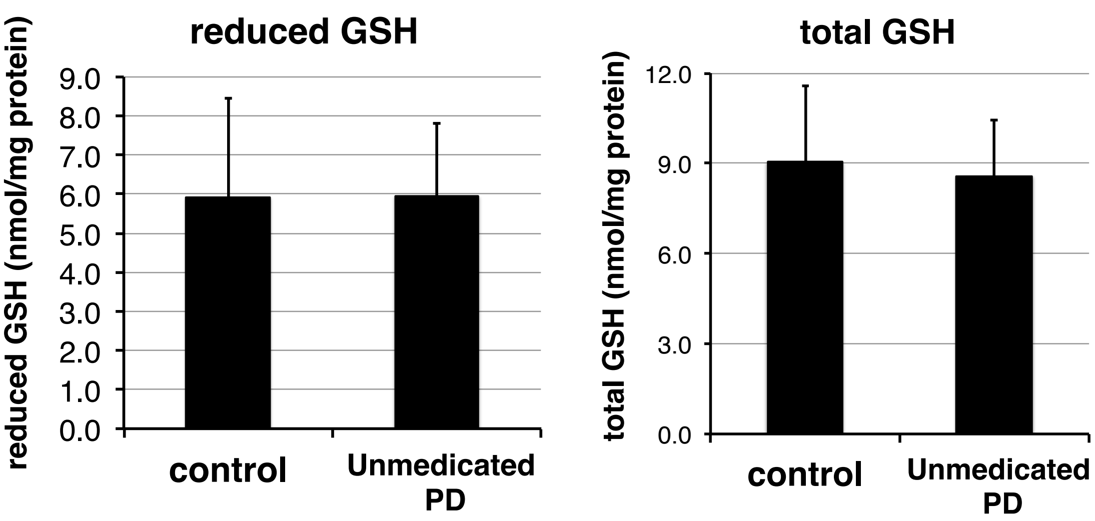

Supplementary Figure S4 | The levels of reduced and total glutathione (GSH) in the erythrocytes of unmedicated PD patients were similar to those of control subjects. The levels of reduced and total glutathione in the erythrocytes of controls (n = 16) and unmedicated patients (n = 14) were determined by using 5,59-dithiobis-(2-nitrobenzoic acid), according to a previously described method.

Title: Oxidation and interaction of DJ-1 with 20S proteasome in the erythrocytes of early stage Parkinson's disease patients

Authors: Yoshiro Saito[1,2], Yoko Akazawa-Ogawa[2], Akihiro Matsumura[3], Kazumasa Saigoh[4], Sayoko Itoh[5], Kenta Sutou[1], Mayuka Kobayashi[1], Yuichiro Mita[1], Mototada Shichiri[2], Shin Hisahara[3], Yasuo Hara[6], Harutoshi Fujimura[7], Hiroyuki Takamatsu[5], Yoshihisa Hagihara[2], Yasukazu Yoshida[2], Takao Hamakubo[8], Susumu Kusunoki[4], Shun Shimohama[3], and Noriko Noguchi[1]

Saito et al., Supplementary Figure
